# Skull X-Ray and CT. Scan in the Management of Head Injuries

**Published:** 1981

**Authors:** D. J. Tawn

**Affiliations:** SHO in Geriatric Medicine, Ham Green Hospital


### Skull X-Ray and CT Scan in the Management of Head Injuries

### Based on work done while a final year student

**D. J. Tawn, MB, ChB**
SHO in Geriatric Medicine, Ham Green Hospital

### ABSTRACT

Accident records of the Frenchay Accident and Emergency Department for the months of January to June 1979 were examined, and 1,534 cases of head injury were recorded. Of these, the numbers of cases submitted to skull x-ray, admitted to hospital and having CT scans were noted. 1,341 skull x-rays were performed of which 23 showed fractures of the cranial vault. 351 patients were admitted, and of these 25 had a CT scan. 12 of these showed injury resulting from the trauma.

### INTRODUCTION

Skull x-rays are traditionally iaken in patients with injuries of the head, mainly to look for fractures of the cranial vault. Occasionally a shift of mid-line structures, e.g. calcified pineal gland, may indicate a space occupying lesion.

The CT scanner is a fairly recent addition to the armoury of investigations which are useful in the management of head trauma. The technique has a diagnostic accuracy approaching 100% in cases of intracranial collections of blood[2,3]. As the procedure is non-invasive, it can be used sequentially in patients slow to recover, or who deteriorate, and its use has also been shown to reduce the frequency of use of more invasive techniques, e.g. arteriography, pneumoencephalography[4,5].

The changes that may be shown by CT are shown in Table 1.

It has been suggested that skull x-ray and CT scanning should be the first neuroradiological procedures and a study has been undertaken to assess how effective this is in practice.

**Table 1**

**Abnormalities Related to Trauma Shown by CT Scan**

*Classification of diseases demonstrable:*

1. Lesions with average absorption value lower than that of brain tissue:
   (a) Oedema of Various degrees
   (b) Chronic subdural haematoma and hygroma
   (c) Post-traumatic atrophic changes
   (d) Obstructive hydrocephalus
   (e) Pneumocephalus
2. Lesions with average absorption value similar to normal brain:
   (a) Centralised diffuse oedema
   (b) Isodense subdural haematoma
3. Lesions with average absorption values greater than normal brain tissue:
   (a) Contusion and laceration
   (b) Heamatomas

### MATERIAL AND METHOD

The records of the Accident Department at Frenchay Hospital were examined for the period of January to June 1979 to ascertain the number of head injury cases seen. Every effort was made to exclude facial injuries alone, where it was thought that there was no possibility of injury to the cranial vault.

After eliciting this information, the numbers of patients who underwent skull x-ray, admission to hospital and CT scanning were noted.

### RESULTS

A total of 1,534 cases of head injury were examined at Frenchay over six months, of which 1,341 (88%) had skull x-rays, 60% of patients were male. 345 of the patients x-rayed were admitted. 193 cases were not x-rayed, of whom 6 were admitted, 180 were discharged and 7 walked out of

**Table 2**

**Head Injury Patients (total 1,534)**

| *Patient Groups* | *Total* | *%* |
|---|---|---|
| Skull X-Ray | 1,341 | 88 |
| No Skull X-Ray | 193 | 12 |
| (a) Admitted | 6 | – |
| (b) Not Admitted | 187 | – |
| (c) Discharged | 180 | – |
| (d) Walked Out | 7 | – |
| Skull X-Ray and Admitted | 345 | 22 |
| Total Admitted | 351 | 23 |

**Table 3**

**Skull X-Rays**

| *Site of Fracture* | *Total* | *%* |
|---|---|---|
| Cranial Vault | 23 | 1.7 |
| Facial Bone | 9 | 0.7 |

the department (Table 2). 23 of the 1,341 skull radiographs showed evidence of fracture to the cranial vault, and a further 9 revealed fractures to facial bones (confirmed by other views). All the cases in which some abnormality was noted in the skull x-ray were admitted (Table 3). 18 (79%) fractures were of the cranial vault and 5 (21%) were of the base of the skull.

There were 351 admissions for head injury during the six month period, involving 349 patients, as 2 were admitted twice. 130 patients (39%) were admitted following road traffic accidents, 175 (53%) were rendered unconscious, 15 (4%) collapsed (6 epileptics, 1 diabetic with hypoglycaemia), and alcohol was noted to be a significant factor in 22 (7%). 17 of the in-patient group had to be excluded from these figures as their notes could not be located (Table 4).

25 of the patients received a CT scan. 11 scans showed evidence of some kind of intracranial haemorrhage, 4 showed brain contusion and 1 showed cerebral oedema. 5 showed cerebral atrophy, not attributable to the head injury. 7 of the patients with a skull fracture had CT scans, of whom 6 showed significant pathology. 8 of the CT scans were normal, and indications for CT scan were mainly neurological. 3 patients showed 2 abnormalities and 1 showed 3 (Table 5).

**Table 4**

**Details of Injury**

| *Incident* | *Subgroup Total* | *Total* |
|---|---|---|
| 1. **Road traffic accidents** | | 130 |
| (a) RTA only | 62 | |
| (b) Rendered unconscious in RTA | 61 | |
| (c) Collapsed at wheel – RTA | 2 | |
| (d) Involved in RTA while drunk | 2 | |
| (e) Rendered unconscious in RTA while drunk | 3 | |
| 2. **Collapsed** | | 8 |
| (a) Hypoglycaemia | 1 | |
| (b) Epileptic fits | 6 | |
| (c) Meningitis | 1 | |
| 3. **Alcohol Induced**[1] | | 16 |
| (a) Fall while intoxicated | 4 | |
| (b) Knocked out in brawl | 12 | |
| 4. **Other incidents**[2] | | 180 |
| (a) Rendered unconscious | 106 | |
| (b) Not rendered unconscious | 74 | |
| TOTAL | | 334[3] |

NB

1. Group does not include those involved in RTAs. Including these brings alcohol related incidents (specified) to 21.
2. This group includes:
   44 children injured while playing
   5 suspected incidents of non-accidental injury
   3 cases of assault
3. 351 admissions less those whose notes could not be found (17), leaving 334.

**Table 5**

**Lesions Seen on CT Scan**

| *Lesion Shown* | *No.* |
|---|---|
| 1. Intracranial haemorrhage | 11 |
| (a) Extradural haematoma | 2 |
| (b) Subdural haematoma | 6 |
| (c) Intracerebral haemorrhage | 3 |
| (d) Subarachnoid haemorrhage | 1 |
| 2. Contusion | 4 |
| 3. Cerebral oedema | 1 |
| 4. Cerebral atrophy (not traumatic) | 5 |
| 5. None | 8 |

### DISCUSSION

There is little doubt that skull x-ray and CT scan of the head are valuable investigations in the management of head injuries[4]. However, decisions must be made as to when and how to use these investigations.

Skull x-ray is a straightforward procedure, but the majority of radiographs do not show a fracture. In this series 88% of the patients were x-rayed and 1.7% of these showed a fracture – a pick up rate of 1 in 60. This means that 1.4% of all the head injuries had a fracture, and 6.5% of the in-patients had a fracture. These results are similar to figures from other areas. In a study by Galbraith[6], skull x-rays revealed a fracture in 9% of patients admitted for head injury. In another study, by Strang[7], 58% of head injury cases were x-rayed, of which 2.7% revealed a fracture (1.5% of all head injury cases), a pick up rate of 1 in 37. 23% of those attending for head injury were admitted, the same as in this study. In figures quoted by Weston[8] for Nottingham, there were 6,300 attendances for head injuries, of which only 30% were x-rayed. Fractures were noted in 2.5% of these, a pick up rate of 1 in 40. There were 921 admissions (15%).

The criteria used for deciding which cases require x-ray or admission are usually left up to the casualty officer. Children and the elderly are usually x-rayed, even with relatively trivial trauma, and all cases where consciousness is lost or clouded should be x-rayed. It must be remembered that a skull fracture is possible with relatively minor trauma, and the presence of a fracture significantly increases the chances of secondary intracranial complications, in particular haematoma and infection.

Many x-rays in head injury cases are done for medico-legal purposes, and some departments now adopt a method where only two views, instead of the usual three, are performed in such cases[9].

CT scanning is also a safe, non-invasive procedure, which can yield extremely valuable information. Fracture of the cranial vault alone is not an indication for CT scan, as many skull fractures do not cause problems. Deterioration in neurological function is a strong indication for CT scan, and a skull fracture in these cases adds weight. Seven of the patients with a skull fracture had CT scans, six of which showed significant intracranial pathology and four were amenable to surgical treatment. If, as these figures suggest, (though the number is small), about 25/% of patients with a skull fracture have significant intracranial damage, then the finding of a skull fracture is very important. Certainly all cases with a skull fracture need admission for close neurological observation. However, the absence of a skull fracture in no way excludes intracranial pathology as 50% of those with significant lesions had no skull fracture.

### CONCLUSION

The value of skull x-ray and CT scanning in head trauma is beyond dispute, but when to use them lies at the discretion of the casualty officer and/or neurosurgeon involved in the patient's care. Skull x-ray should be performed on all cases where the trauma was severe enough to cause loss or clouding of consciousness. Such cases need admission for observation. CT scans are reserved for cases in which there is either slow progress, or a deterioration in neurological status.

### REFERENCES

1. MERINO DE VILLASANTE, J. and TAVERAS, J. M. (1976). 'Computerised Tomography in Acute Head Trauma', *American Journal of Roentgenology,* **126**, 767-778.
2. DUBLIN, A. B., FRENCH, B. N. and RENNICK, J. M. (1977). 'Computerised Tomography in Head Trauma', *Radiology,* **122**, 365-369.
3. GALBRAITH, S., TEASDALE, G. and BLAIKLOCK, C. (1976). 'Computerised Tomography of Acute, Traumatic, Intracranial Haematoma: Reliability of Neurosurgeons Interpretations', *British Medical Journal*, iv, 1371-1373.
4. AMBROSE, J., GOODING, M. R. and UTTLEY, D. (1976). 'EMI scans in the Management of Head Injurie', *Lancet,* i, 847-849.
5. ZIMMERMAN, R. A. et al. (1978). 'Cranial Computed Tomography in Diagnosis and Management of Acute Head Trauma', *American Journal of Roentgenology,* **131**, 27-34.
6. GALBRAITH, S. et al. (1977). 'Head Injury Admissions to a Teaching Hospital', *Scottish Medical Journal,* **22**, 129-132.
7. STRANG, I. et al. (1978). 'Head Injuries in Accident and Emergency Departments at Scottish Hospitals', *Injury,* **10**, 154-159.
8. WESTON, P. A. M. (1978). 'Skull X-ray Policy' (letter), *Lancet,* ii, 792.
9. MACKINTOSH, C. E. (1979). 'Casualty X-rays', *British Journal of Hospital Medicine,* **22** (3), 296.

### ACKNOWLEDGMENTS

I would like to express my sincere thanks to Dr. W. D. Jeans and Dr. G. Thomson for all their help and encouragement in the preparation of this paper.

